# Draft Genome Sequence of *Vibrio* sp. Strain Evh12, a Bacterium Retrieved from the Gorgonian Coral *Eunicella verrucosa*

**DOI:** 10.1128/genomeA.01729-15

**Published:** 2016-02-11

**Authors:** Telma Franco, Gianmaria Califano, Ana C. S. Gonçalves, Catarina Cúcio, Rodrigo Costa

**Affiliations:** a Centre of Marine Sciences (CCMar), Microbial Ecology and Evolution Research Group, University of Algarve (UAlg), Gambelas, Faro, Portugal; b Department of Biological Sciences, State University of Santa Cruz-UESC, Ilhéus, Brazil; c Microbial Systems Ecology, Department of Aquatic Microbiology, Institute for Biodiversity and Ecosystem Dynamics, University of Amsterdam, Amsterdam, The Netherlands

## Abstract

To shed light on the associations established between *Vibrio* species and soft corals in coastal ecosystems, we report here the draft genome sequence of *Vibrio* sp. strain Evh12, a bacterium that has been isolated from the gorgonian coral *Eunicella verrucosa* and that shows antagonistic activity against *Escherichia coli*.

## GENOME ANNOUNCEMENT

*Vibrio* species are widespread, predominantly marine, planktonic, and/or symbiotic bacteria with potential pathogenic behavior (1–3). Of emerging concern are the roles that opportunistic *Vibrio* spp. may play in disease incidence and coral reef decline in a changing climate (4). In contrast with the wealth of information available on *Vibrio* spp. inhabiting calcified corals in tropical latitudes (3, 5, 6), associations established by *Vibrio* bacteria and so-called soft corals, such as gorgonians (Octocorallia, Gorgoniidae), which are also widespread corals across temperate biomes (7), remain understudied. To advance our understanding of the genetic features underlying host colonization and persistence, and of the putative commensal versus pathogenic behavior of gorgonian-associated *Vibrio* spp., we report here the draft genome sequence of *Vibrio* sp. strain Evh12, isolated from the gorgonian coral *Eunicella verrucosa*.

*E. verrucosa* was collected at ca. 15 m depth off the coast of Sagres, Portugal (37 0′ 31.82′“N; 8 55” 29.60′’ W), and immediately transported to the laboratory (8). Strain Evh12 was isolated on marine agar medium using standard procedures (9) after 7 days of incubation at 19°C (8). Whole-genome DNA extraction and sequencing were carried out as described by Gonçalves et al. (10). The sequence output was 644 Mb, corresponding to ca. 118× coverage of the genome. Sequence reads were assembled *de novo* into 17 contigs with the NGen DNA assembly software by DNAStar, Inc. Gene prediction and annotation were performed with the Rapid Annotations using Subsystems Technology (RAST) prokaryotic genome annotation server, version 2.0 (11). The genome is 5,472,963 bp long, with a G+C content of 44%. It possesses 4,799 coding sequences (CDSs), 108 tRNAs, and 24 rRNAs. *Vibrio* sp. Evh12 presents the highest 16S rRNA gene similarity (99.8%) with *Vibrio gigantis* LGP 13^T^ (12), isolated from the oyster *Crassostrea gigas*, being closely related to *Vibrio* sp. Vb278 (99.4% 16S rRNA gene similarity), an antagonistic bacterium cultured from a marine sponge (9, 10). Strain Evh12 displays a versatile nutrient assimilation profile, comprising 501 genes for carbohydrate metabolism, with 26 genes involved in chitin and *N*-acetylglucosamine utilization. Among these, we detected chitin-binding protein (CBP)- and catabolic sensor kinase (*chiS*)-encoding genes, which are required for chitinase expression (13), along with three chitinase biosynthetic genes. Extending its nutrient-foraging capacity is the complete gene cluster *pvsABCDE* for vibrioferrin (siderophore) biosynthesis and transport (14). The predicted chemotactic ability of strain Evh12 is supported by the vast repertoire of *fli* and *flh* genes required for flagellar protein biosynthesis, including motor switch proteins FliM and FliN, and the complete set of Che protein-coding genes *cheABRVWYZ*, which coordinate the transfer of chemoreceptor signals to flagellar motor components. Strain Evh12 displays high genome-wide conservation with *Vibrio* sp. Vb278, thus sharing the pivotal symbiosis and virulence factors reported for Vb278 (10); this strengthens the notion of *Vibrio* spp. as generalist and opportunistic commensals capable of populating multiple hosts.

### Nucleotide sequence accession numbers.

The genome sequence of *Vibrio* sp. Evh12 has been deposited in the European Nucleotide Archive (ENA) (http://www.ebi.ac.uk/ena) under the accession numbers FAUO01000001 to FAUO01000017. The study identification number is PRJEB10717.
